# Emotional Reactivity and Behavioral Problems in Preschoolers: The Interplay of Parental Stress, Media-Related Coping, and Child Screen Time

**DOI:** 10.3390/children12020188

**Published:** 2025-02-05

**Authors:** Roma Jusienė, Rima Breidokienė, Edita Baukienė, Lauryna Rakickienė

**Affiliations:** Institute of Psychology, Faculty of Philosophy, Vilnius University, Universiteto 9/1, 01513 Vilnius, Lithuania; rima.breidokiene@fsf.vu.lt (R.B.); edita.baukiene@fsf.vu.lt (E.B.); lauryna.rakickiene@fsf.vu.lt (L.R.)

**Keywords:** preschool age, emotional reactivity, screen-based media, parental distress, behavioral problems

## Abstract

**Background/Objectives:** Excessive screen time has been linked to poorer developmental outcomes in preschool years, with the impact depending on context. Parents of emotionally reactive children, in particular, may use screens to manage their child’s emotions, especially when experiencing distress themselves. This reliance on screens can hinder the development of self-regulation, leading to behavioral difficulties. Our study aimed to explore how emotional reactivity, parental distress, screen time, and media-related coping interact while contributing to externalizing (behavioral) problems in preschool-aged children. **Methods:** The study included 754 children (49.1% girls), aged 2–5 years (M = 44.6 months, SD = 13.7). Parents reported children’s emotional reactivity and externalizing problems using the Child Behavior Checklist and provided data on daily screen time and media-related coping behaviors. Bivariate correlations, group comparisons, and structural equation modeling were employed to analyze the data. **Results:** Children averaged 111.86 min of daily screen time (SD = 83.94), with 35.2% of parents using screens as a coping strategy. Emotional reactivity was the strongest predictor of externalizing problems, while the role of screen time was weak, yet significant. Parental distress was positively associated with child emotional reactivity and screen time. Structural equation modeling showed that child emotional reactivity related to behavioral problems both directly and indirectly via parental distress, media-related coping, and increased screen time. **Conclusions:** The findings highlight emotional reactivity as a key factor in child behavioral problems, emphasizing the cumulative risks posed by parental distress and screen use. The results suggest interventions promoting healthier screen habits and supporting parental well-being.

## 1. Introduction

A growing body of research links excessive screen-based media use and externalizing problems in early childhood, although the effect sizes of these associations are usually small [1,2]. It is commonly agreed, that not only the quantity, but also the quality of screen time is important in terms of outcomes, be it content or circumstances of screen use [3]. There is some evidence that screen time may be particularly detrimental when used as a tool to manage children’s behavior or emotions [4,5,6]. Although screens can provide short-term relief for both children and parents, excessive reliance on this strategy may hinder the development of self-regulation and reinforce dysregulated behaviors. The present study aims to explore the complex interplay between child temperament, parental distress, and screen use in contributing to externalizing problems during the preschool years.

### 1.1. Screen Exposure in Early Childhood

Preschool-aged children today are immersed in screen-based activities, with digital devices becoming integral to their daily routines. While some studies have demonstrated benefits of limited exposure to interactive, educational, and age-appropriate screen-based activities for cognitive development [7,8], extended or unsupervised screen use poses certain risks. Parents are recommended to limit screen time to 1 h a day for children aged 2 to 5 [9] as prolonged exposure to screens has been associated with poorer child development [10,11] and in particular with delayed language development in preschool age [12,13,14], attentional difficulties [15], obesity, and other health problems [16]. Also, empirical evidence links longer screen time with increased behavioral challenges [1], although the direction of these associations is not always clear. Many studies have found links between screen time and subsequent externalizing problems [11,17]; however, the findings become less clear when background problems are considered. A recent longitudinal study found that greater externalizing problems at age 3 were linked to a moderate rise in screen time two years later, but not vice versa [18]. This supports the idea that screens are potentially being employed as a means to manage challenging behaviors or to calm dysregulated children during early childhood. These dynamics underscore the need to examine the underlying mechanisms linking screen time and behavioral difficulties, particularly in the context of child temperament and parental stress.

### 1.2. Child Emotional Reactivity

Child temperament, a construct encompassing individual differences in reactivity and regulation, is a key factor in understanding behavioral challenges. Reactivity refers to physiological, emotional, and attentional responses to stimuli, while regulation involves the processes that modulate these responses [19]. Effortful control, a regulatory mechanism, gradually matures during early childhood, enabling children to manage their emotions and behaviors more effectively over time [20]. However, this regulatory capacity is not fully developed in preschool-aged children, leaving them more dependent on caregivers for emotional support.

Highly reactive children, who often struggle with self-soothing and regulation, rely even more heavily on caregivers for comfort [21]. Thus, temperament may act as a key factor for child’s behavior, including behavioral challenges, which are further influenced by parental responses to specific behaviors [20,22]. Furthermore, temperament characteristics and environmental factors both interact in child developmental outcomes, either increasing or decreasing the risk of various difficulties or disorders. Temperament-based regulation can mitigate risk factors and stressors [19,22].

Researchers suggest there is a link between child emotional reactivity and screen time [23]. Children with low self-regulatory abilities are more difficult to soothe or manage, which may prompt parents to rely more heavily on digital media as a regulatory strategy, thereby increasing the child’s screen exposure. Although this approach may offer short-term relief, it can hinder the development of self-regulation over time [24,25], further exacerbating behavioral difficulties.

### 1.3. Parental Stress

Parental stress is another critical factor influencing both children’s screen time and behavioral outcomes. Research suggests that parents with limited personal, social, and financial resources and are not able to meet their child care needs, are more prone to expose children to screen use [26,27]. Greater young children’s exposure to screens has been shown to relate to the poorer relational well-being of mothers [28], parental conflict [29], socioeconomic issues [4], and overall parenting stress [27,30,31]. Along these lines, parents, experiencing more stress in any domain of their life, may loosen screen time rules and limits, allowing more usage to ease their own stress or manage their child’s dysregulation. Thus, screens can become a coping tool for parenting challenges [31].

### 1.4. The Interplay of Individual and Contextual Factors in Explaining Externalizing Behaviors

The association between parental stress and screen use may be particularly pronounced in families raising temperamentally reactive children. Parents of reactive children may experience heightened stress due to the children’s difficulties with self-regulation and a tendency towards emotional outbursts. These challenges can lead parents to use screens as a tool to soothe their child or to create a temporary reprieve from parenting demands [32]. To sum up, the relationship between screen time, child temperament, and externalizing problems is complex and bidirectional. While longer screen time has been linked to greater behavioral challenges [1], it may also reflect parents’ attempts to manage pre-existing difficulties [32]. For example, parents of children with high emotional reactivity may use screens as a regulatory tool, inadvertently reinforcing dysregulated behaviors and increasing the child’s reliance on screens for emotional management. Moreover, parental stress interacts with child temperament to shape media-related decisions [4]. Parents who experience high levels of stress may be less consistent in enforcing screen time limits, particularly when managing a reactive child. These dynamic highlights the interplay between individual and environmental factors in contributing to externalizing problems.

Although screen time is by no means the sole contributor to behavioral challenges, it may serve as an important contextual factor that interacts with child and parental characteristics [4,27,30,33]. The complexity of the interplay between screen time, children’s factors, such as emotional reactivity and gender, and parental factors, such as parental distress and the media-related coping practices, are still under-researched in preschoolers, and is therefore the main aim of the present study.

The following research questions for this study were established: (1) How are child screen time and parental media-related coping associated with preschoolers’ behavioral problems? (2) What are the relationships among child emotional reactivity, parental distress, and child screen time, and how do these factors interact to predict behavioral problems in preschoolers? (3) Do child screen time and parental media-related coping contribute directly or indirectly to the prediction of preschoolers’ behavioral problems, accounting for child emotional reactivity and parental distress?

## 2. Materials and Methods

### 2.1. Participants and Procedure

The study sample included 754 children (49.1% girls), aged 2 to 5 years old, not having chronic diseases, with 95.5% of children attending pre-school education services (kindergarten). The mean children’s age was 44.6 months (SD = 13.7). The baseline sociodemographic features of participants are presented in Table 1. These characteristics correspond to the main tendencies of educational attainment, marital status, and place of residence in Lithuania.

This research is a part of an extensive national study “Electronic Media Use and Young Children’s Health” conducted in the year 2017 and funded by the Research Council of Lithuania (agreement no. GER-006/2017). The research was approved by the Regional Biomedical Research Ethics Committee of Vilnius (No. 158200-2017/04). Parents of toddlers and pre-school aged children living in various regions of Lithuania were invited to take part in the research through pre-school education institutions, health care specialists, and social media. After signing the informed consent, parents completed paper–pencil or online questionnaires.

### 2.2. Instrument and Measures

Child emotional reactivity and externalizing problems were measured using the Lithuanian version of the Child Behavior Checklist [2,34,35]. The CBCL/1½-5 is a 100-item parent-report measure designed to screen for the problem behaviors in preschool-age children, and consists of 7 scales: emotionally reactive, anxious/depressed, somatic complaints, withdrawn, sleep problems, attention problems, and aggressive behavior. The first four scales are then combined to create the internalizing problems factor, whereas attention problems and aggressive behavior comprise the externalizing problems factor. Each item describes a specific behavior, and parents rate its frequency on a three-point Likert-type scale (0—not true; 1—somewhat or sometimes true; 2—very true or often true) based on the preceding 2 months.

The Emotional reactivity scale consists of 9 items and measures a child’s propensity for intense and frequent emotional reactions as well as difficulty adapting to changes [36]. The total score on the scale can range from 0 to 18. A higher score indicates more pronounced emotional reactivity in children. The internal consistency of the scale in this study was appropriate, indicating its good reliability (Cronbach’s alpha = 0.72).

The Externalizing problems scale consists of 24 items and measures a child’s behavioral problems. The internal consistency of the scale in this study was appropriate, indicating its good reliability (Cronbach’s alpha = 0.87).

Child screen time was measured using a parent-report questionnaire. Parents provided information about the duration of their child’s screen time on weekdays and weekends, for various types of screen-based devices (smartphone, tablet, computer, game console, and TV). They answered separate questions about the usual daily duration for each device, selecting from the following options: “no or almost no usage” (1); “15 min to 30 min per day” (2); “30 min to 1 h per day” (3); “1 to 2 h per day” (4); “2 to 3 h per day” (5); “3 to 4 h per day” (6); “more than 4 h per day” (7). To estimate the average daily screen time, each response was first converted to minutes as follows: (1)—0 min; (2)—22.5 min; (3)—45 min; (4)—90 min; (5)—150 min; (6)—210 min; (7)—270 min. The average daily screen time was then calculated using the formula: (screen use on weekdays [converted to minutes] × 5 days + screen use on weekends [converted to minutes] × 2 days)/7 days. A higher score indicates longer screen time. The screen time measure was developed based on common practices and is widely used in studies examining screen time behaviors in children (e.g., 2, 7, 20). The inclusion of separate questions for different devices and times of the week was designed to improve accuracy and data quality and reduce the possible effects of bias, underestimation, and overestimation.

Parental distress was measured using a six-item scale, where the respondent parent (caregiver) rated each item on a 5-point scale (from 1 (‘almost every day’) to 5 (‘rarely or never’)) based on how often they had experienced the following in the past six months: (1) physical pain or discomfort (e.g., headache, stomach pain, etc.); (2) sadness or depressive mood; (3) irritability or bad mood; (4) nervous tension or anxiety; (5) problems falling asleep; (6) drowsiness or lack of activity. A total distress score was calculated, and for the analysis, the variable was transformed so that higher values indicated greater parental distress. The reliability of this scale in the present study was very good (Cronbach’s alpha = 0.83).

Parental media-related coping was measured on a 4-point scale (1—never or almost never; 2—sometimes; 3—often; 4—almost always) on how often the respondent parent (caregiver) uses screens when a child is upset or frustrated. In a further analysis, this variable was transformed into a binary pseudo-variable—if parents use screens to calm down a child (0—never or almost never; 1—at least sometimes).

### 2.3. Statistical Analysis

Bivariate Spearman correlations among study variables were calculated. Mean comparisons between two groups were counted using a Student *t*-test for interval variables and a non-parametric Mann–Whitney U test for ordinal variables. Effect sizes (Cohen’s d) were calculated to assess the magnitude of the differences between groups. To control for the risk of Type I error due to multiple comparisons, the Bonferroni correction was applied while adjusting the significance level by dividing the original significance threshold (α = 0.05) by the number of tests. Correlations, descriptive statistics, distributions, and comparisons were run in SPSS 23.0. The Mplus 6.0 software package [36] was used for structural equation modeling. The following model fit indices were utilized: the Root Mean Square Error of Approximation (RMSEA), the Comparative Fit Index (CFI), and the Tucker–Lewis Index (TLI). A good model fit is indicated by an RMSEA value of <0.05 and CFI and TLI values > 0.90.

## 3. Results

The main characteristics of the participants are presented in Table 1. According to the results of the study, the average duration of children’s screen time, as indicated by their parents, was 111.86 min (*SD* = 83.94 min). Additionally, 35.23% (*n* = 260) of the respondent parents reported at least sometimes using screens when their child was upset, frustrated, or sad. Those parents who completed online questionnaires (*n* = 164, 21.8%) had a higher educational level (Mann–Whitney U = 36147.00, *p* < 0.001) and reported higher parental distress (t(749) = −4.45, *p* < 0.001), lower child emotional reactivity (t(749) = 2.39, *p* = 0.018), and a shorter child screen time (t(749) = 2.48, *p* = 0.013) than parents who completed paper–pencil questionnaires (*n* = 587, 78.2%). There were no differences between these groups in the level of child externalizing problems, child gender, and parental media-related coping.

First, correlations between child variables (age, emotional reactivity, screen time, and externalizing problems) and parental distress were analyzed (see Table 2). Since the majority of the mothers shared similar sociodemographic characteristics in terms of educational level and family status (80.1% had a higher education, either university or non-university type, and 82.4% were married), these variables were not controlled in the study assuming that there may not be enough variability to influence the outcomes.

The results indicated that child externalizing problems were associated with emotional reactivity, screen time, and parental distress (see Table 2). Child externalizing problems were most strongly correlated with emotional reactivity (large effect), followed by parental distress (medium effect). The relationship with screen time was small but significant. Additionally, child emotional reactivity was positively related to both screen time and parental distress, suggesting that, according to parents, more emotionally reactive children spent more time using screens and exhibited higher levels of externalizing problems. These relationships were significant, but small in effect size. Furthermore, parents of more emotionally reactive children reported experiencing greater distress. Emotional reactivity correlation with parental distress was small but significant. The results also revealed a positive, but small correlation between parental distress and child screen time. Finally, child age shows mostly weak or negligible correlations with other variables, with a small but significant positive correlation with screen time.

Secondly, we examined whether the study variables differed based on parental media-related coping. The results, presented in Table 3, indicate that children whose parents used screens as a coping strategy were younger, more emotionally reactive, exhibited more externalizing problems, and spent more time using screens. Additionally, parents who relied on media-related coping reported higher levels of distress.

To control for the risk of Type I error due to multiple comparisons, the Bonferroni correction was applied. With five tests performed, the original significance threshold (α = 0.05) was adjusted to α = 0.01. As shown in Table 3, the differences in emotional reactivity, screen time, parental distress and externalizing problems remained significant after the correction, while differences in child age did not meet the corrected threshold. Regarding the effect sizes, presented in Table 3, child emotional reactivity, externalizing problems, and parental distress show statistically significant but small differences between groups. In contrast, screen time had a moderate effect size, suggesting a more meaningful difference between the groups.

Thirdly, we examined gender differences in the study variables. The results showed that girls and boys differed only in their levels of externalizing problems, with boys having a higher mean score for externalizing problems than girls (see Table 4). However, after applying the Bonferroni correction, this difference did not meet the corrected threshold (α = 0.01). There were no gender differences in parental media-related coping (χ^2^ = 0.52, *p* = 0.538)

**Figure 1:**
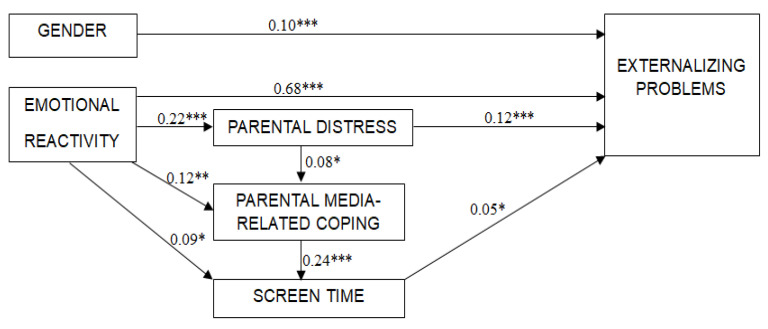
Structural equation model (standardized regression coefficients are indicated). Note. *** *p* < 0.001; ** *p* < 0.01; * *p* < 0.05.

Finally, a model was developed to explore the interrelationships between child externalizing problems, screen time, parental distress, and parental media-related coping. The initial model was constructed based on the correlations and group comparisons presented in Table 2, Table 3 and Table 4. Later, the model was adjusted by removing statistically insignificant relationships and adding new statistically significant relationships. The final model fits perfectly with the data: χ^2^ (6) = 2.98, *p* = 0.812, CFI = 1.00, TLI = 1.01, RMSEA = 0.000. Child age was controlled in the model (see Figure 1).

The results, as depicted in the model, showed that child externalizing problems were directly predicted by higher emotional reactivity, greater screen time, higher parental distress, and male gender (R^2^ = 0.53). Child screen time was predicted by child emotional reactivity and parental media-related coping. Additionally, parental media-related coping was predicted by child emotional reactivity and parental distress. Although parental coping did not directly predict child externalizing problems, it had an indirect effect through increased child screen time.

## 4. Discussion

Although some evidence suggests direct links between screen media use and behavior problems in early childhood, findings across studies remain inconsistent [1,30]. This highlights the need to investigate potential mediating factors. The present study aimed to examine the interplay among child temperament, parental distress, and screen use in contributing to externalizing problems during the preschool years.

Firstly, the results of our study revealed that child gender, emotional reactivity, parental distress, and screen time all play roles in predicting child’s externalizing problems, with emotional reactivity having the most significant direct effect. This suggests that these variables together account for a substantial portion of the variability in externalizing problems (R^2^ = 0.53). Screen time had a significant but small direct effect on externalizing problems, when accounting for other individual and contextual variables. This suggests that while screen time contributes to externalizing problems, its effect is weaker compared to other predictors. These findings align with previous studies that report significant but weak associations between screen time and externalizing behavior problems [1,2]. Additionally, consistent with prior research, the results support the notion that the relationship between screen time and externalizing problems is influenced by other background variables, such as male gender [1] and parental stress [37]. A recent illustration of the interplay between parental mental health, screen practices, and child outcomes can be seen in the natural experiment of the COVID-19 pandemic, during which there was a significant increase in screen time, as well as in parental stress and child externalizing behaviors [33].

Secondly, while parental media-related coping did not directly predict externalizing problems, its effect was mediated by increased child screen time. This aligns with the effect size results: externalizing problems show statistically significant but small differences between parents who used media-related coping and those who did not. In contrast, screen time demonstrates a moderate effect size, indicating a more substantial difference between the groups.

Although the effect sizes were small, both child emotional reactivity and parental distress contributed to the variance in parental media-related coping, suggesting these variables play a meaningful role in understanding this behavior. In line with the aforementioned studies, parents of highly reactive children may be more likely to adopt coping strategies that are immediately effective in soothing a child, such as increased screen time. Moreover, child emotional reactivity may be a primary or additional driver of parental distress and distressed parents may struggle to consistently enforce rules or find alternative emotion regulation strategies, leading to more reliance on screens. Over time, excessive screen time may exacerbate emotional reactivity and disrupt self-regulation, contributing to the development of externalizing behaviors [24,25].

Thirdly, the results of our study suggest that child emotional reactivity is a central variable, influencing multiple factors such as parental distress, media-related coping, and screen time, and directly and indirectly affecting externalizing problems. Beyond the direct effect of emotional reactivity on behavioral problems, parental distress, and screen time may serve as mediators, helping to explain these associations. Direct, indirect, and interactive effects of child temperament (both reactivity and regulation) together with other contextual and relational factors associated with children’s adjustment problems are well-grounded in many previous studies [22]. Although the contribution of temperamental characteristics to children’s successful everyday functioning in modern digital environment still needs further research attention.

To sum up, the results of our study reveal that emotional reactivity, as a potential factor of vulnerability, plays a significant role in behavioral problems. It also contributes to parental distress and to the use of screens as a coping mechanism, which in turn predicts behavioral problems. These findings highlight the importance of emotional reactivity, particularly when other unfavorable factors in the emotional environment of the family are present. Thus, cumulative family emotional risks are crucial in understanding children’s behavioral problems. Given that children’s self-regulation abilities are not yet fully developed, their reactivity may predominate [20]. Therefore, emotional reactivity, as an innate aspect of a child’s temperament, plays a critical role in the regulation and expression of their behavior. Additionally, as toddlers and preschool children have less developed self-control, they are more susceptible to the negative influences of their home environment [38]. Parents must therefore be mindful of their emotional reactions—managing their own distress—and avoid using screens to calm their children or themselves.

### Limitations and Future Directions

This study analyzed a large, population-based sample of toddlers and preschoolers, using statistical analyses that considered interactions and pathways among variables. However, there are several limitations to acknowledge. First, child emotional reactivity and behavioral problems, screen time, parental distress, and media-related coping were all reported by parents. While parents and caregivers can provide valuable insights into a child’s traits and their surrounding environment, their perceptions may be influenced by bias and subjectivity, particularly in relation to the child’s behavioral issues and emotional reactivity. Additionally, parental coping was measured using a single item assessing screen use to calm a child. This measure did not capture the various situations and contexts in which screens are used as a coping strategy, which may limit the reliability of the analysis. We suggest that future research should use more comprehensive measures of screen-based parental coping. Moreover, when reporting on screen-based media use, social desirability bias is a significant factor to consider. Although the study sample aligns well with the demographic profile of the national population, it primarily includes families with low to moderate socioeconomic risks and children without severe health concerns. Finally, it is important to mention the data collection strategy. Most parents of participating children were reached through doctor’s offices and educational institutions and completed a paper-and-pencil questionnaire. However, approximately one-fifth of the study sample was reached via social media, and these parents completed an online questionnaire. Some demographic and study variables differed between these two groups, suggesting that the method of participant recruitment and data collection may have influenced the results. While the dual nature of the data collection could be considered a methodological limitation, we believe it helped mitigate sampling bias, which is unavoidable in research relying on convenience sampling.

Furthermore, our study focused solely on one aspect of temperament—reactivity. However, the other dimension, regulation, is also crucial for understanding both behavioral problems and the potential effects of screen-based media use. Future research should adopt multimethod approaches (e.g., passive sensing apps and questionnaires) and longitudinal designs to assess the effects of screen-based media use on child outcomes. Additionally, it is important for future studies to examine the screen time of the entire family and explore the possible cumulative effects of both children’s and parents’ total screen time on child development [13,39].

## 5. Conclusions

Despite the limitations of our study, we conclude that a child’s emotional reactivity, as one dimension of temperament, along with parental distress, child screen time, and parental media-related coping, collectively contribute to less favorable conditions for the child’s psychosocial development. These findings suggest potential directions for interventions aimed at reducing or managing behavioral problems. While individual predispositions, such as temperament traits, typically cannot be directly altered, managing behavioral issues may be facilitated by establishing appropriate, child-friendly screen media use.

Based on the results of this study, we emphasize the need to educate both parents and professionals working with families about children’s mental health and the responsible use of screen media. It is crucial to provide guidance, support, and training for parents, particularly those raising children with higher emotional reactivity. A key priority is to educate parents on establishing healthy screen time habits for their children. This includes (1) managing the frequency and duration of screen time based on the child’s age; (2) carefully selecting age-appropriate content—such as games, movies, and videos—and using parental control tools to manage unsuitable content; and (3) ensuring that screen time does not replace essential childhood activities, such as physical activity and free play, which are vital for development [39].

## Figures and Tables

**Table 1:** Baseline characteristics of the participants.

Characteristics	(*n* = 754)
Child age (months) ^a^	44.6 (*SD* = 13.7)
Attending pre-school education services	
%Yes	95.5 (*n* = 720)
% No	4.5 (*n* = 34)
Child gender	
% Girls	49.1 (*n* = 370)
% Boys	50.9 (*n* = 384)
Child’s mother’s mean age (years)	32.7 (*SD* = 4.2)
Parental education	
% Higher university education	65.9 (*n* = 497)
% Higher non-university education	14.2 (*n* = 107)
% Secondary professional education	12.9 (*n* = 97)
% Secondary non-professional or less	7.1 (*n* = 53)
Parental marital status	
% Married	82.4 (*n* = 621)
% Cohabitation or single-parent household	17.6 (*n* = 133)
Family residence	
% Urban (major city)	61.9 (*n* = 464)
% Rural (district center, small town, village)	38.1 (*n* = 290)
Child’s average screen time (min)	111.86 (*SD* = 83.94)
Parental use of screens when their child was upset, frustrated, or sad	
% Never or almost never	64.77 (*n* = 494)
% At least sometimes	35.23 (*n* = 260)

Note: ^a^ Values are presented in means and standard deviations (*SD*).

**Table 2:** Bivariate correlations among child and parental variables.

Study Variable	1.	2.	3.	4.
1. Child age	-			
2. Emotional reactivity	−0.04	-		
3. Screen time	0.12 **	0.08 *	-	
4. Externalizing problems	−0.04	0.71 **	0.11 **	-
5. Parental distress	0.03	0.21 **	0.10 **	0.26 **

Note. * *p* < 0.05; ** *p* < 0.01.

**Table 3:** Comparison of study variables according to parental media-related coping.

Variables	Do Not Use (*n* = 478)	Use (*n* = 260)	T	df	*p*	Cohen’s d	95% CI (Lower)	95% CI (Upper)
	*M* (*SD*)	*M* (*SD*)						
Child age	45.58 (13.42)	43.03 (14.17)	2.41	736	0.016	−0.19	−0.34	−0.04
Emotional reactivity	2.70 (2.32)	3.41 (2.52)	−3.85	736	<0.001	0.30	0.15	0.45
Screen time	97.15 (70.15)	138.29 (98.74)	−5.95	404.19	<0.001	0.51	0.35	0.66
Externalizing problems	11.33 (6.53)	13.43 (6.98)	−4.08	736	<0.001	0.31	0.16	0.47
Parental distress	12.13 (4.65)	13.22 (5.18)	−2.94	736	0.003	0.26	0.07	0.38

**Table 4:** Comparison of study variables according child gender.

Variables	Girls (*n* = 370)	Boys (*n* = 384)	T	df	*p*	Cohen’s d	95% CI (Lower)	95% CI (Upper)
	*M* (*SD*)	*M* (*SD*)						
Child age	44.81 (13.47)	44.48 (13.93)	0.324	752	0.746	−0.024	−0.17	0.12
Emotional reactivity	2.99 (2.52)	2.92 (2.30)	0.413	752	0.680	−0.029	−0.17	0.11
Screen time	111.90 (84.72)	111.82 (83.28)	0.013	752	0.990	−0.001	−0.14	0.14
Externalizing problems	11.46 (6.89)	12.61 (6.53)	−2.35	752	0.019	0.171	0.03	0.31
Parental distress	12.48 (4.97)	12.52 (4.79)	−0.10	752	0.923	0.008	−0.14	0.15

## Data Availability

The data presented in this study are available on request from the corresponding author due to ethical and confidentiality reasons.
